# Supplementary material for: SPACA6 ectodomain structure reveals a conserved superfamily of gamete fusion-associated proteins
Source: Commun Biol. 2022 Sep 17;5:984. doi: 10.1038/s42003-022-03883-y (PMC9482655; doi:10.1038/s42003-022-03883-y)
Supplement: Supplementary file 2 — Supplementary Information [file 42003_2022_3883_MOESM2_ESM.pdf]

Supplementary Information for
SPACA6 ectodomain structure reveals a conserved superfamily of gamete fusion-associated proteins

Tyler D.R. Vance, Patrick Yip, Elisabet Jiménez, Sheng Li, Diana Gawol, James Byrnes, Isabel Usón, Ahmed Ziyyat, and Jeffrey E. Lee*.

Correspondence: Jeffrey E. Lee
Email: : jeff.lee@utoronto.ca

This PDF file includes:

Figures S1 to S19
Tables S1

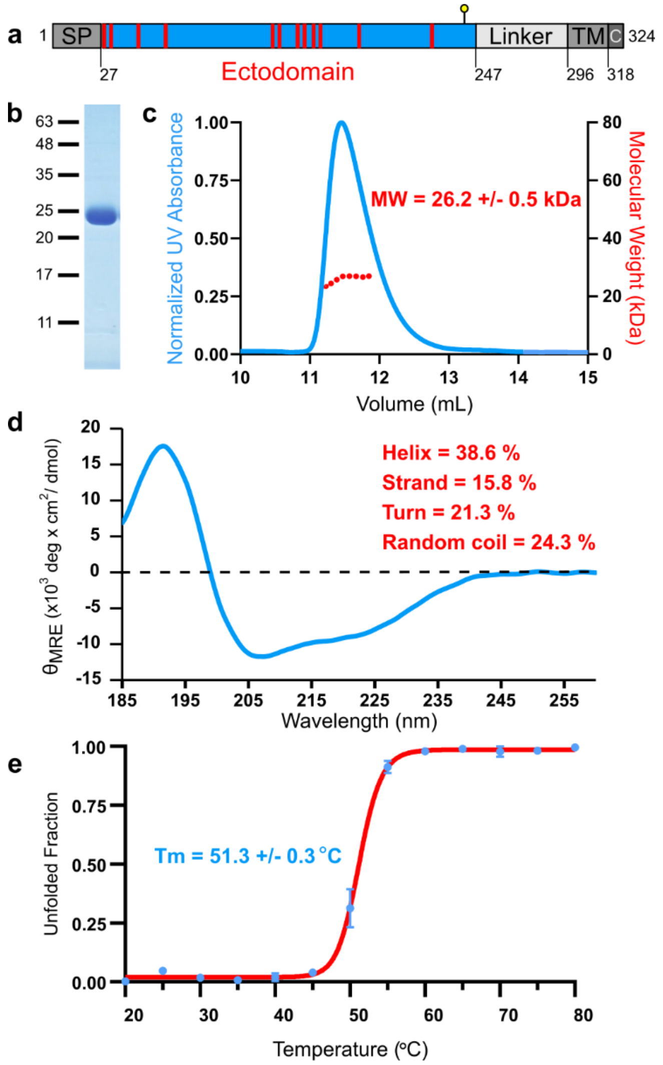

Figure S1. SPACA6 ectodomain expresses as a folded, monomeric protein. a Domain architecture of full-length SPACA6. The signal peptide (SP), transmembrane helix (TM), and cytoplasmic tail (C) are indicated in the schematic. Red lines indicate cysteine residues, and yellow lollipop indicates predicted N-linked glycosylation site. **b** Coomassie-stained SDS-PAGE analysis of purified recombinant SPACA6 ectodomain. Molecular weights based on protein ladder bands are shown on the left. **c** SEC-MALS analysis of recombinant SPACA6 ectodomain. Size-exclusion chromatogram with normalized UV absorbance (280 nm) is shown in the blue curve with calculated molecular weight values derived from light scattering shown in red dots. The presented molecular weight is the average of calculated molecular weights from each individual light-scattering measurements (*n*=108) taken within the peak, ± standard deviation. **d** Far-UV CD spectra of recombinant SPACA6 ectodomain at 0.16 mg mL^{-1}. Spectra is an average of ten accumulations that are buffer corrected and smoothened. **e** Thermal melt of recombinant SPACA6 ectodomain. Mean residue ellipticity was recorded at 207 nm. Values are averages of independent triplicates (*n=3*) with error bars for standard deviation shown in blue.

>SPACA6_Native
MALLALASAVPSALLALAVFRVPAWACLLCFTTYSERLRICQMFVGMRSPKLEECEEAFTAAFQG
LSDTEINYDERSHLHDTFTQMTHALQELAAAQGSFEVAFPDAAEKMKKVITQLKEAQACIPPCGL
QEFARRFLCSGCYSRVCDLPLDCPVQDVTVTRGDQAMFSCIVNFQLPKEEITYSWKFAGGGLRTQ
DLSYFRDMPRAEGYLARIRPAQLTHRGTFSCVIKQDQRPLARLYFFLNVTGPPPRAETELQASFR
EVLRWAPRDAELIEPWRPSLGELLARPEALTPSNLFLLAVLGALASASATVLAWMFFRWYCSGN

>SPACA6_Recombinant
MKLCILLAVVAFVGLSLGCLLCFTTYSERLRICQMFVGMRSPKLEECEEAFTAAFQGLSDTEINY
DERSHLHDTFTQMTHALQELAAAQGSFEVAFPDAAEKMKKVITQLKEAQACIPPCGLQEFARRFL
CSGCYSRVCDLPLDCPVQDVTVTRGDQAMFSCIVNFQLPKEEITYSWKFAGGGLRTQDLSYFRDM
PRAEGYLARIRPAQLTHRGTFSCVIKQDQRPLARLYFFLNVTGGRLVPRGSHHHHHHHHHH

>SPACA6_Recombinant_Processed
CLLCFTTYSERLRICQMFVGMRSPKLEECEEAFTAAFQGLSDTEINYDERSHLHDTFTQMTHALQ
ELAAAQGSFEVAFPDAAEKMKKVITQLKEAQACIPPCGLQEFARRFLCSGCYSRVCDLPLDCPVQ
DVTVTRGDQAMFSCIVNFQLPKEEITYSWKFAGGGLRTQDLSYFRDMPRAEGYLARIRPAQLTHR
GTFSCVIKQDQRPLARLYFFLNVTGGRLVPR

Figure S2. Sequences of native SPACA6 and recombinant SPACA6 ectodomain. One-letter amino acid sequences for SPACA6 Native, Recombinant, and the Recombinant construct after secretion and tag-removal with thrombin. Orange indicates amino acids of the four-helix bundle; green indicates hinge region amino acids; blue indicates Ig-like domain amino acids; grey indicates native signal peptide; red indicates native transmembrane helix; black indicates non-ectodomain native sequences; grey highlight indicates BiP signal peptide for *Drosophila* S2 cell secretion; green highlight indicates thrombin cleavage site; black highlight indicates 10x-His tag; and purple indicates putative N-linked glycosylation site.

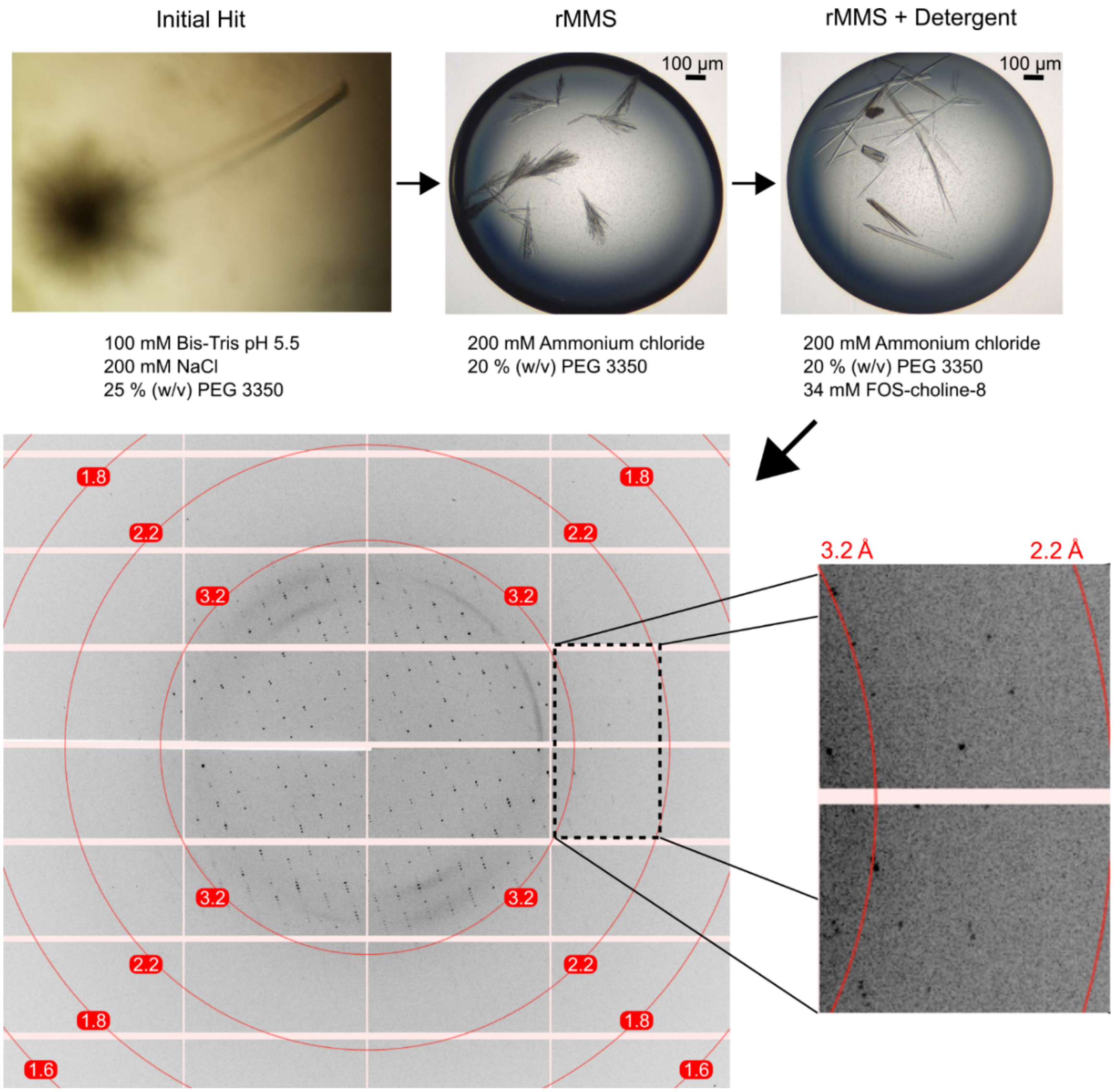

Figure S3. Crystal growth and diffraction of SPACA6. Upper images were taken during optimization; Optimization of SPACA6 crystals (upper images) with the compositions of the reservoir solutions stated the images. Upper left: Initial crystals obtained by vapor-diffusion; these crystals diffracted poorly. Upper middle: Crystals obtained when initial crystals were used as seeds for random microseed matrix screening (rMMS). Upper right: Larger crystals obtained through screening detergents as additives. Lower left: Diffraction image produced from the detergent-stabilized crystals, collected at the NE-CAT (24-ID-C) beamline at the Advanced Photon Source (Argonne National Laboratory, Lemont, IL). Red circles indicate (from innermost to outermost) 3.2 Å, 2.2 Å, 1.8 Å, and 1.6 Å resolution rings. Lower right insets: Higher magnification of boxed region showing high-resolution reflections to 2.2 Å resolution.

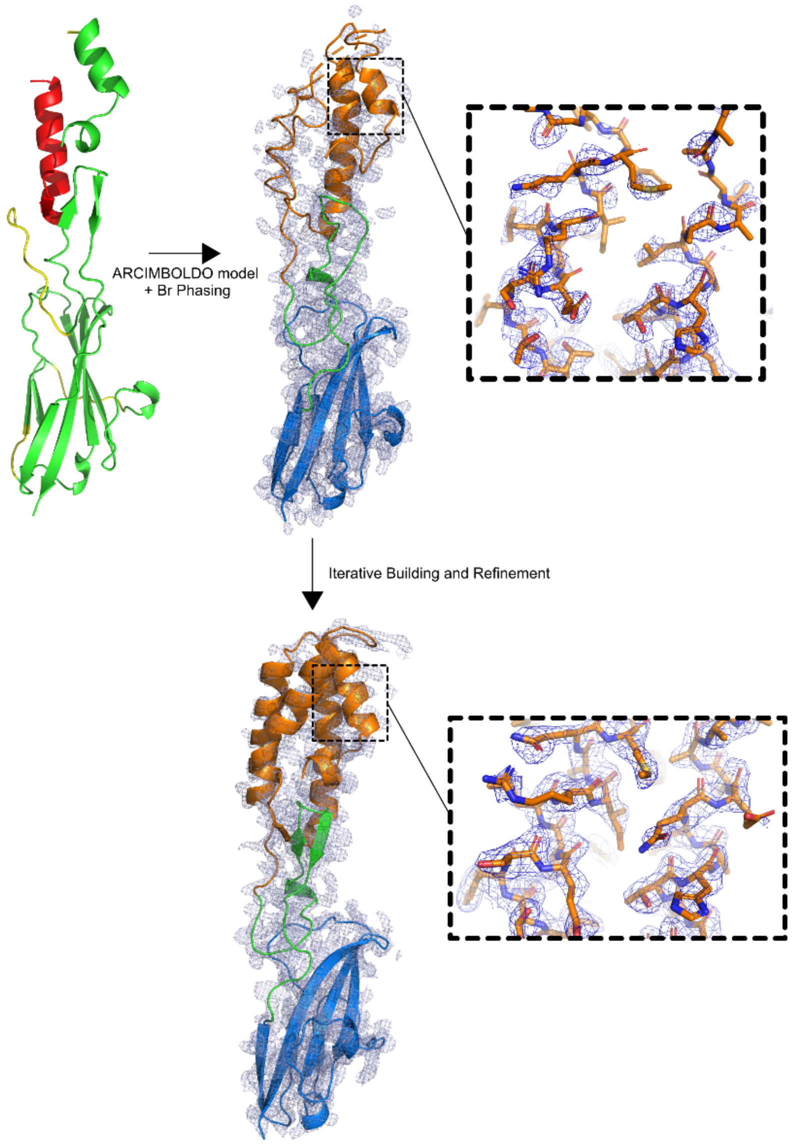

Figure S4. Initial ARCIMBOLDO_SHREDDER model and electron density map. Upper left: Initial SPACA6 model produced via ARCIMBOLDO_SHREDDER (65% complete). Portions of the model built into strong electron density are colored green; portions of the model built into strong electron density but modeled as poly-alanine are colored yellow; and portions of the model unconnected to other parts and made up of poly-alanine are colored red. Upper right: Following Br-SAD phasing and ARCIMBOLDO_SHREDDER molecular replacement, an initial phased combined experimental electron density map ($|F_o|$) map shown at 1σ was obtained. The inset shows the poor electron density of the 4HB. Lower: Following iterative building/refinement, the final electron density map ($2|F_o|-|F_c|$) at 1σ superimposed with the refined SPACA6 structure is shown. The inset shows the same region of the 4HB as the upper inset. Structures are colored orange for 4HB, green for hinge, and blue for Ig-like domain.

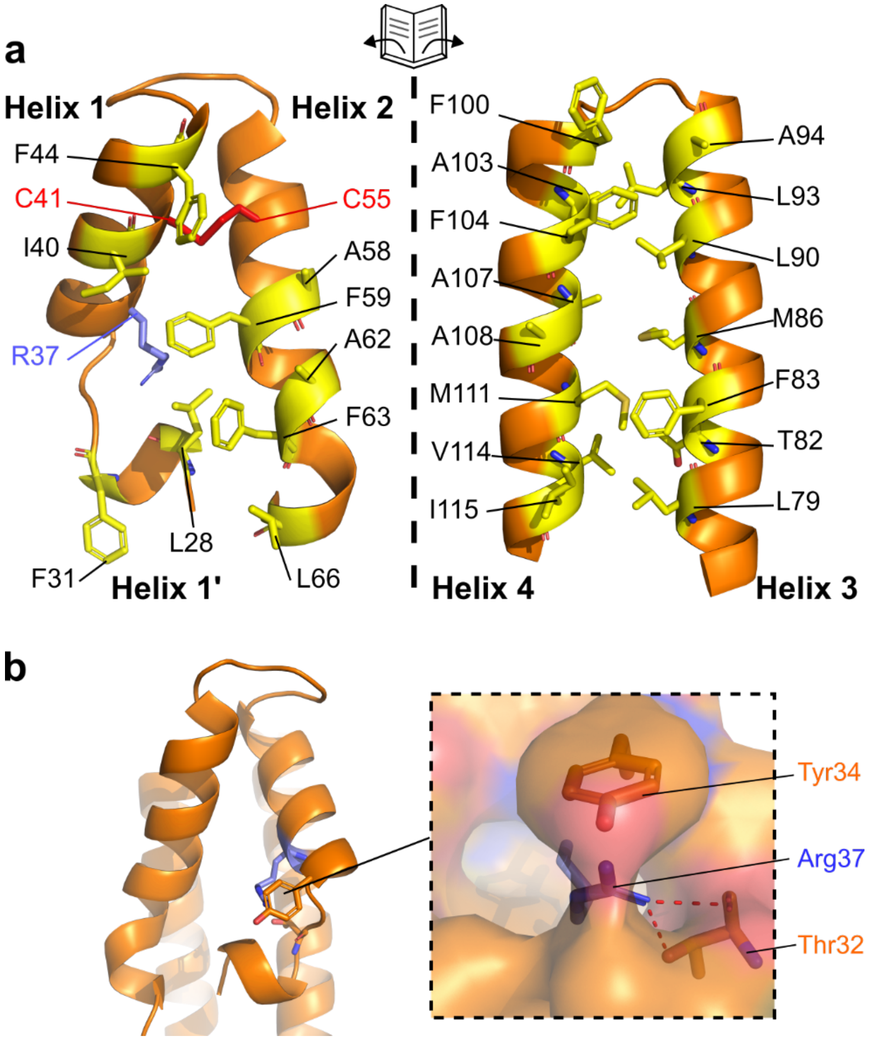

Figure S5. Conserved arginine within 4HB hydrophobic core. a Cross-section of SPACA6 four-helix bundle. The bundle was split in the middle and opened to reveal the hydrophobic core. Hydrophobic components of the 4HB core are colored yellow and displayed as sticks. Components of the 4HB core include Arg37 (purple) and a Cys41-Cys55 disulfide bond (red). **b** Triangular face of the 4HB, with Tyr34, Arg37 (purple), and Thr32 shown as sticks. Inset shows the Arg37 interaction with Thr32 and its occlusion by Tyr34.

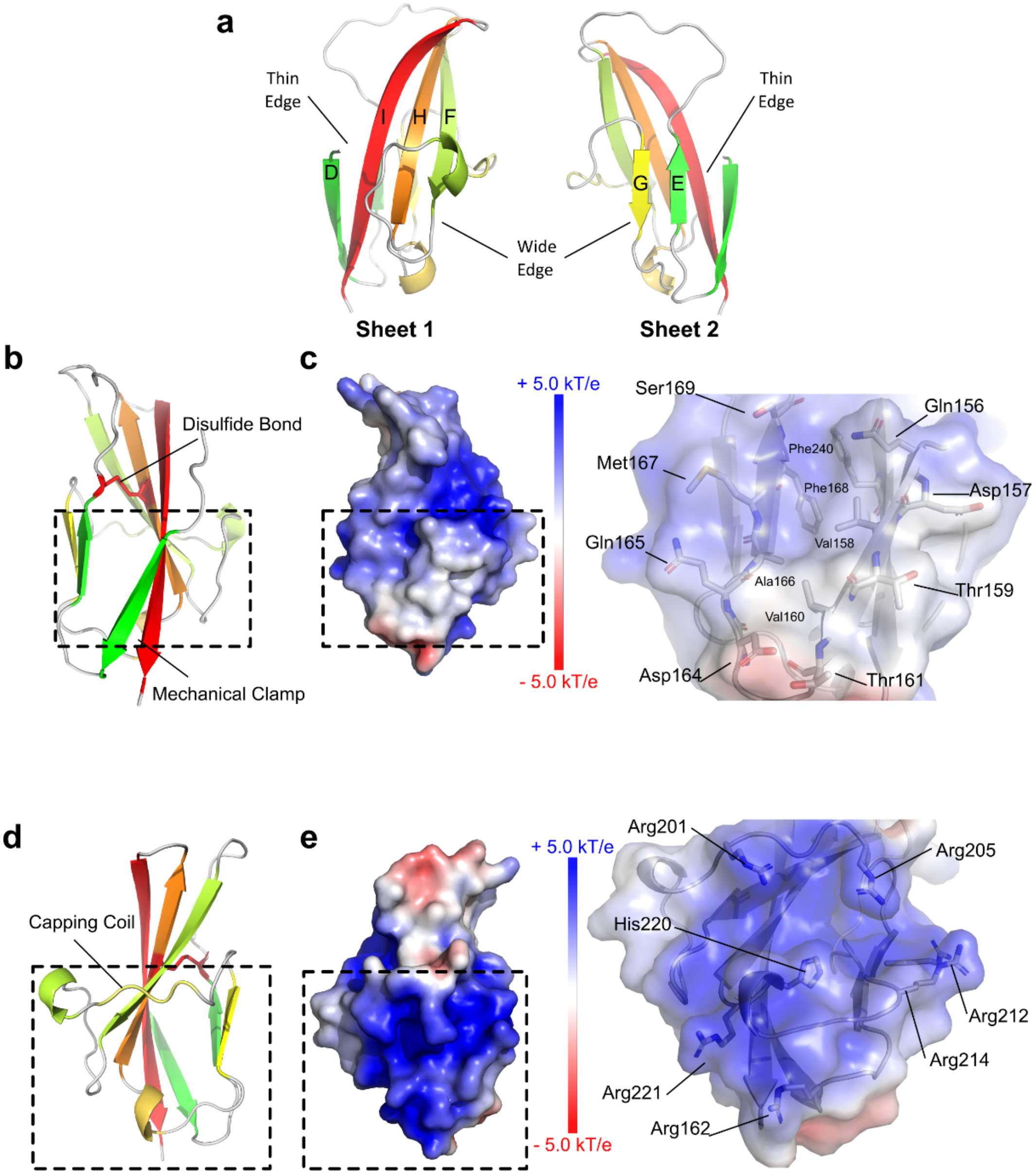

Figure S6. C-terminal Ig-like domain of SPACA6. a Ribbon diagram of the SPACA6 Ig-like domain with its six strands labelled. **b** Ribbon diagram of the thinner edge of the Ig-like domain. The disulfide bond is shown as sticks. **c** Surface electrostatic potential of the thinner edge of the Ig-like domain. Hydrophobic patch outlined by dashed box is shown as a zoomed image (right) with the residues shown as sticks. **d** Ribbon diagram of the wider edge of the Ig-like domain. Capping coil that covers the hydrogen bonds in Strand F is shown. **e** Surface electrostatic potential of the wider edge of the Ig-like domain. Positively charged cavity outlined by dashed box is shown as a zoomed image (right) with labeled residues shown as sticks.

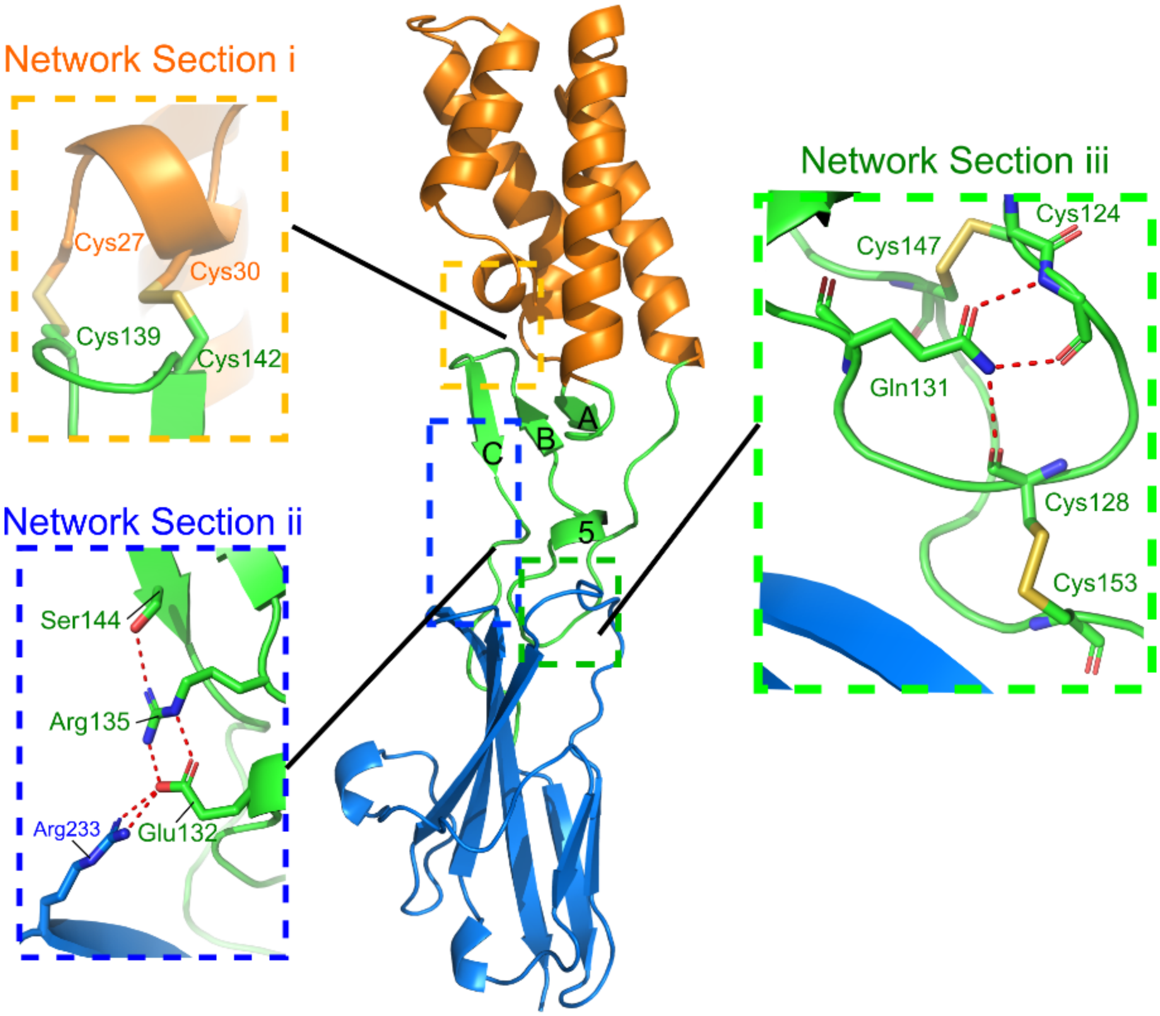

Figure S7. SPACA6 hinge region connects the 4HB and Ig-like domains. Ribbon diagram of the hinge region (green), which connects the 4HB (orange) and the Ig-like domain (blue). The three sections of the covalent/electrostatic network that rigidifies the region are shown in detail.

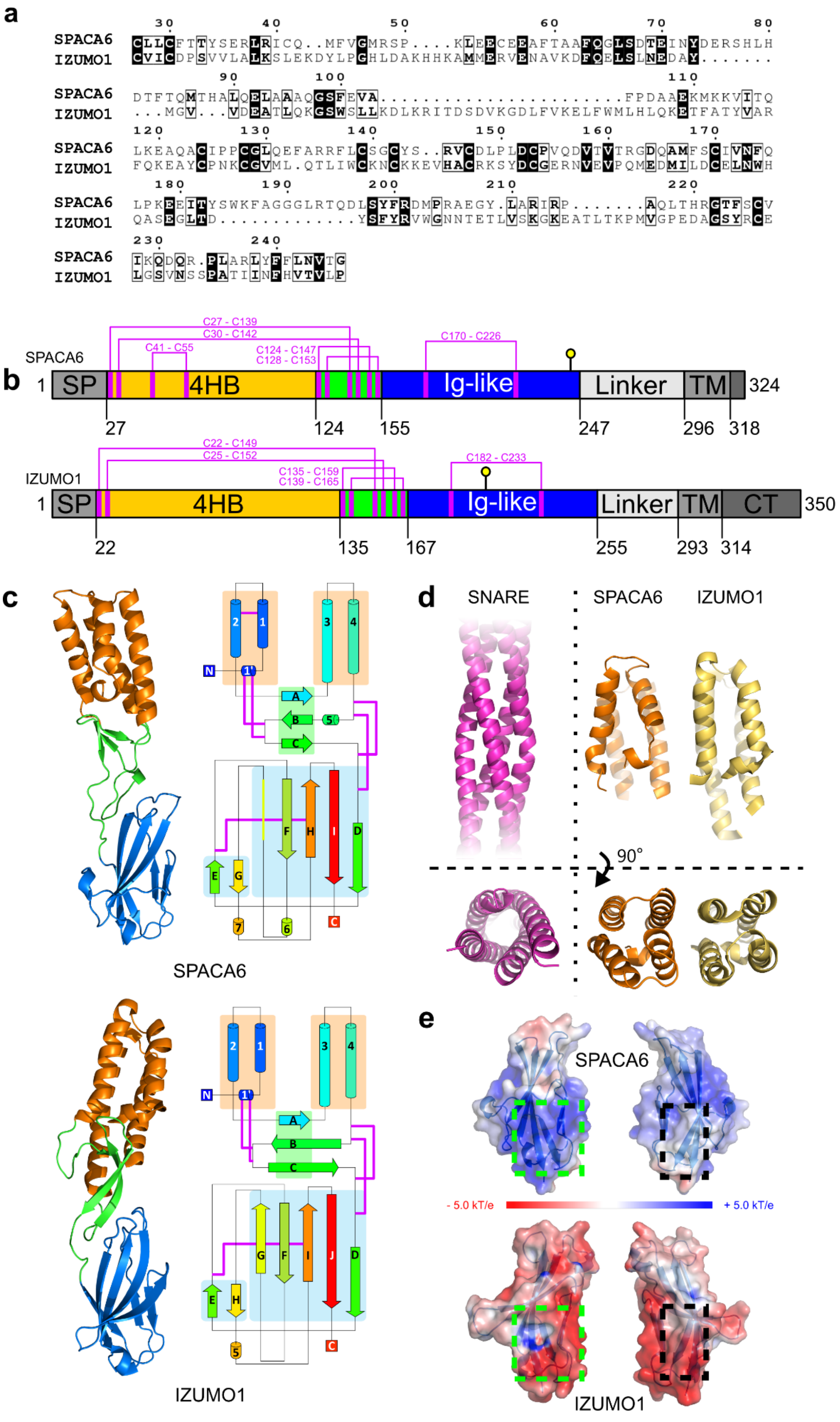
a
SPACA6 CLLCFTTYSERLRICQ..MFVGMRSP....KLEECEEAFTAAFQGLSDTEINYDERSHLH
IZUMO1 CVICDPSVVLALKSLEKDYLPGHLDAKHHKAMMERVENAVKDFQELSLNEDAY.......
SPACA6 DTFTQMTHALQELAAAQGSFEVA.......................FPDAAEKMKKVITQ
IZUMO1 ...MGV...VDEATLQKGSWSLLKDLKRITDSDVKGDLFVKELFWMLHLQKETFATYVAR
SPACA6 LKEAQACIPPCGLQEFARRFLCSGCYS..RVCDLPLDCPVQDVTVTRGDQAMFSCIVNFQ
IZUMO1 FQKEAYCPNKCGVML.QTLIWCKNCKKEVHACRKSYDCGERNVEVPQMEDMILDCELNWH
SPACA6 LPKEEITYSWKFAGGGLRTQDLSYFRDMPRAEGY.LARIRP.......AQLTHRGTFSCV
IZUMO1 QASEGLTD.............YSFYRVWGNNTETLVSKGKEATLTKPMVGPEDAGSYRCE
SPACA6 IKQDQR.PLARLYFFLNVTG
IZUMO1 LGSVNSSPATIINFHVTVLP
b
SPACA6
C27 - C139
C30 - C142
C41 - C55
C124 - C147
C128 - C153
C170 - C226
SP 4HB Ig-like Linker TM
1 27 124 155 247 296 318 324
IZUMO1
C22 - C149
C25 - C152
C135 - C159
C139 - C165
C182 - C233
SP 4HB Ig-like Linker TM CT
1 22 135 167 255 293 314 350
c
SPACA6
IZUMO1
d
SNARE
SPACA6
IZUMO1
90°
e
SPACA6
IZUMO1
- 5.0 kT/e
+ 5.0 kT/e

Figure S8. SPACA6 and IZUMO1 share a similar structure. a Human SPACA6 (RefSeq: NP_001303901) and IZUMO1 (RefSeq: NP_872381) ectodomain sequences aligned using CLUSTAL OMEGA. Strictly conserved residues are displayed as white text on a black background. Residues with conservative substitutions are in bold. **b** Domain architecture schematic of SPACA6 and IZUMO1. Magenta lines indicate disulfide bonds and yellow lollipop indicates a predicted N-linked glycan site. Abbreviations: SP, signal peptide; TM, transmembrane helix; CT, cytoplasmic tail. **c** Ribbon and topology diagrams comparing ectodomain structures for SPACA6 and IZUMO1 (PDB: 5F4E). Domains are colored according to panel b. **d** Four-helix bundle comparison in two orientations. Ribbon diagrams for autophagic SNARE complex (PDB: 4WY4, magenta), SPACA6 (orange), and IZUMO1 (PDB: 5F4E, yellow). **e** Surface electrostatic potential of SPACA6 and IZUMO1 Ig-like domains. The positively charged pocket from SPACA6 is outlined with a dashed green line, and the hydrophobic surface from SPACA6 is outlined with a dashed black line.

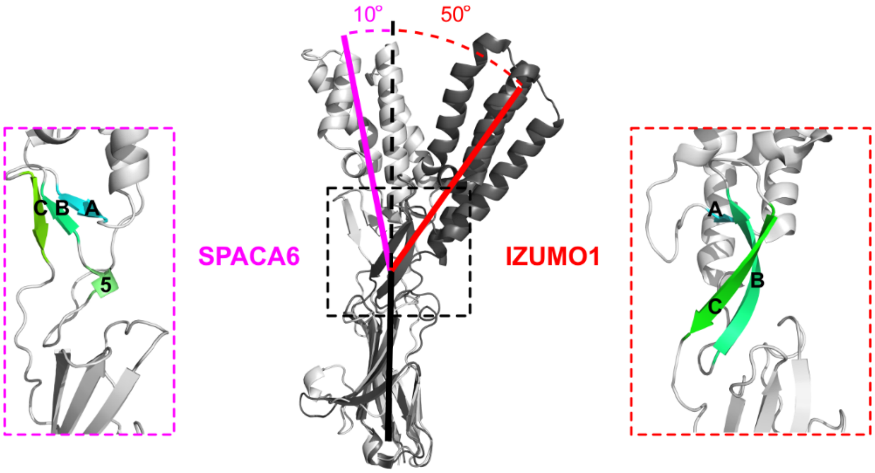

Figure S9. Differences in domain orientation between IZUMO1 and SPACA6. SPACA6 (white) and IZUMO1 (PDB: 5F4E, black) structures were aligned by the Ig-like domain to compare the orientation of the 4HB. Insets show the hinge region for both SPACA6 (purple box) and IZUMO1 (red box).

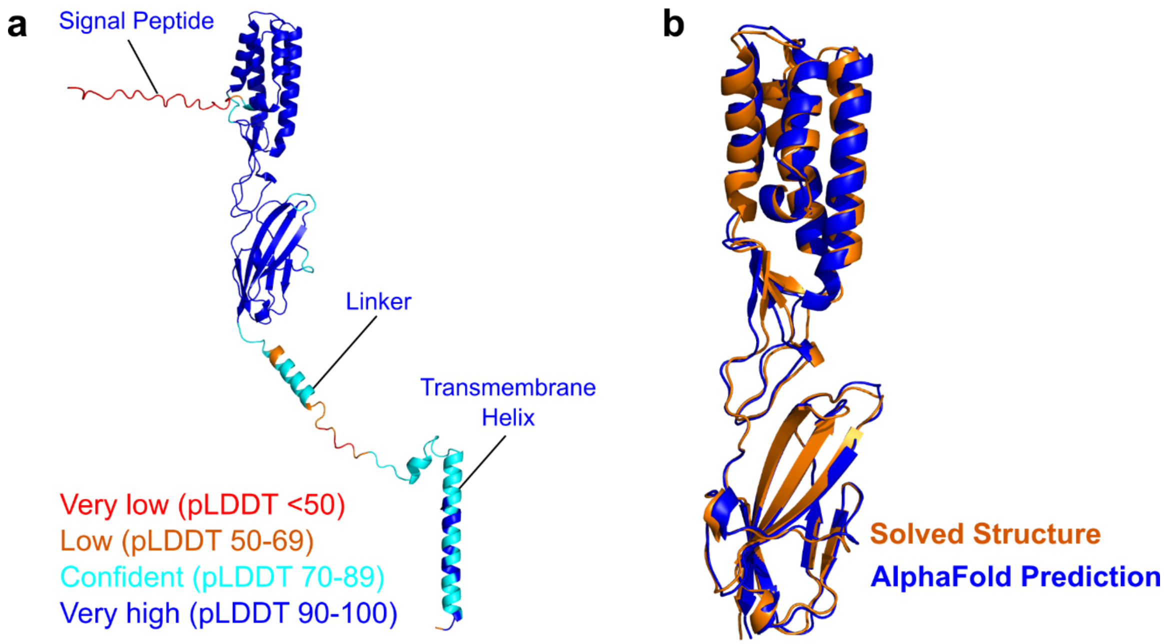

Figure S10. AlphaFold prediction of SPACA6. a Full-length SPACA6 prediction from AlphaFold (AF-W5XKT8-F1) colored by confidence level (pLDDT). **b** Ectodomain comparison between the solved structure (orange) and the AlphaFold prediction (blue) of SPACA6. Structures align with an RMSD of ~1.1 Å (aligning 1494 atoms).

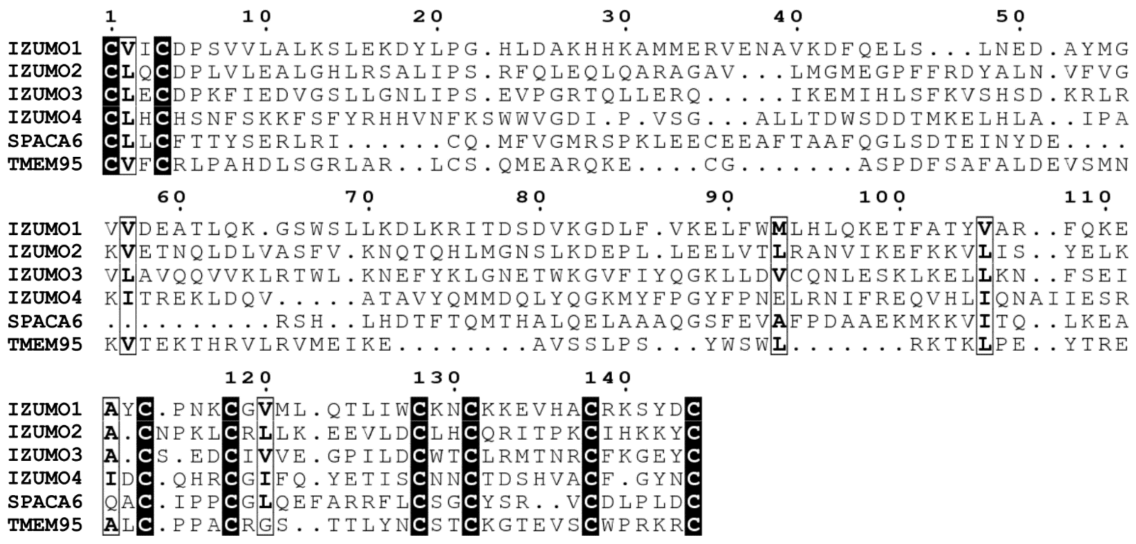

Figure S11. Sequence alignment of the 4HB domains from the proposed IST-superfamily. The 4HB domains from human IZUMO1 (NCBI: NP_872381), IZUMO2 (NCBI: NP_689571), IZUMO3 (NCBI: NP_001351937), IZUMO4 (NCBI: NP_001034935), SPACA6 (NCBI: NP_001303901), and TMEM95 (NCBI: XP_016880054) were aligned using CLUSTAL OMEGA. Strictly conserved residues are displayed as white text on a black background. Residues with conservative substitutions are in bold. Alignment numbering corresponds to the human IZUMO1 sequence.

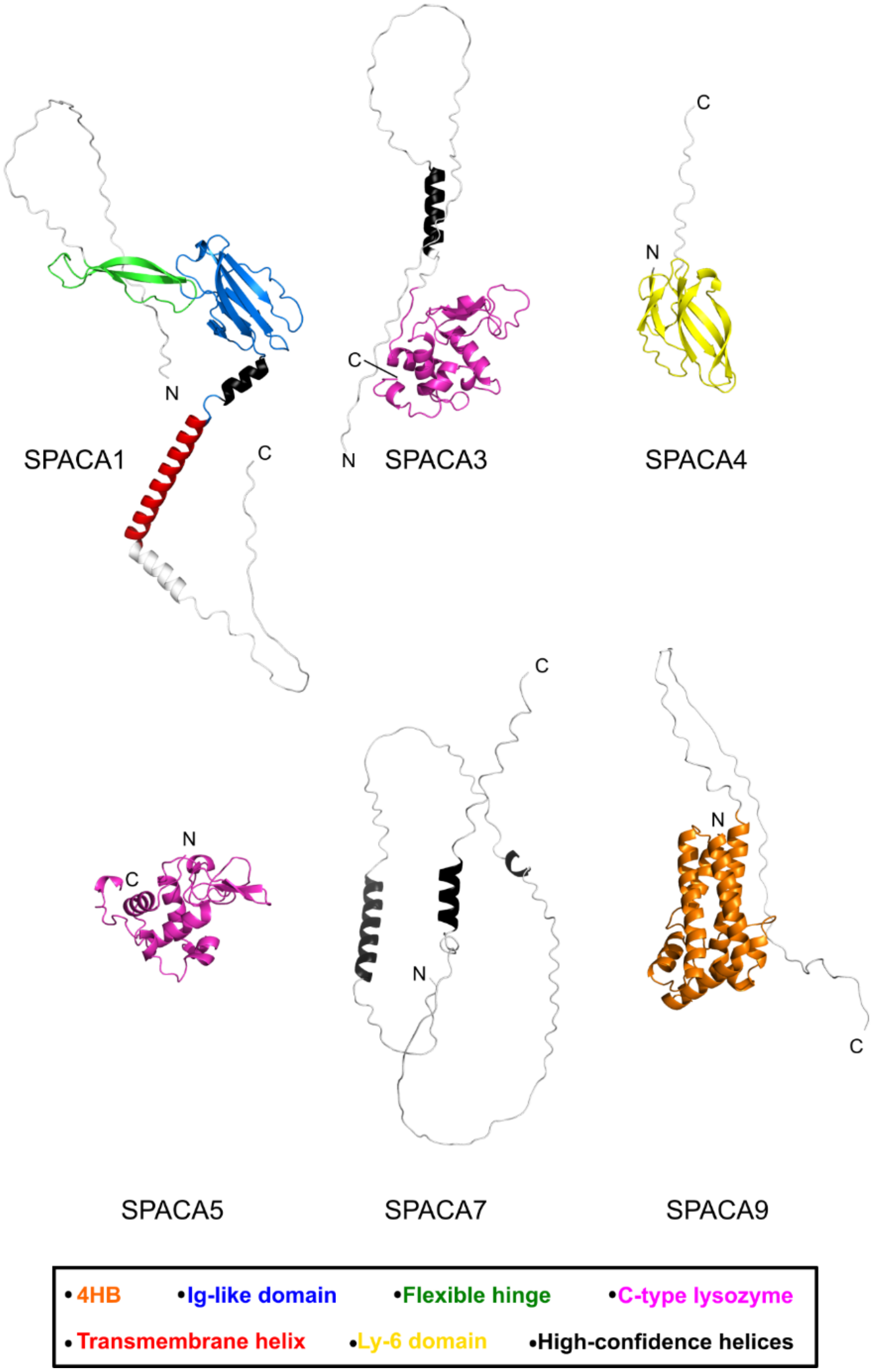

Figure S12. AlphaFold predictions of structures of SPACA proteins. Cartoon representations of AlphaFold structure predictions of human SPACA1 (AF-Q9HBV2-F1), SPACA3 (AF-Q8IXA5-F1), SPACA4 (AF-Q8TDM5-F1), SPACA5 (AF-Q96QH8-F1), SPACA7 (AF-Q96KW9-F1), and SPACA9 (AF-Q96E40-F1). Black helices are non-interacting with high predictive confidence. Areas of low predictive confidence are colored white. Signal peptides predicted for SPACA1, SPACA4, SPACA5, and SPACA7 were removed.

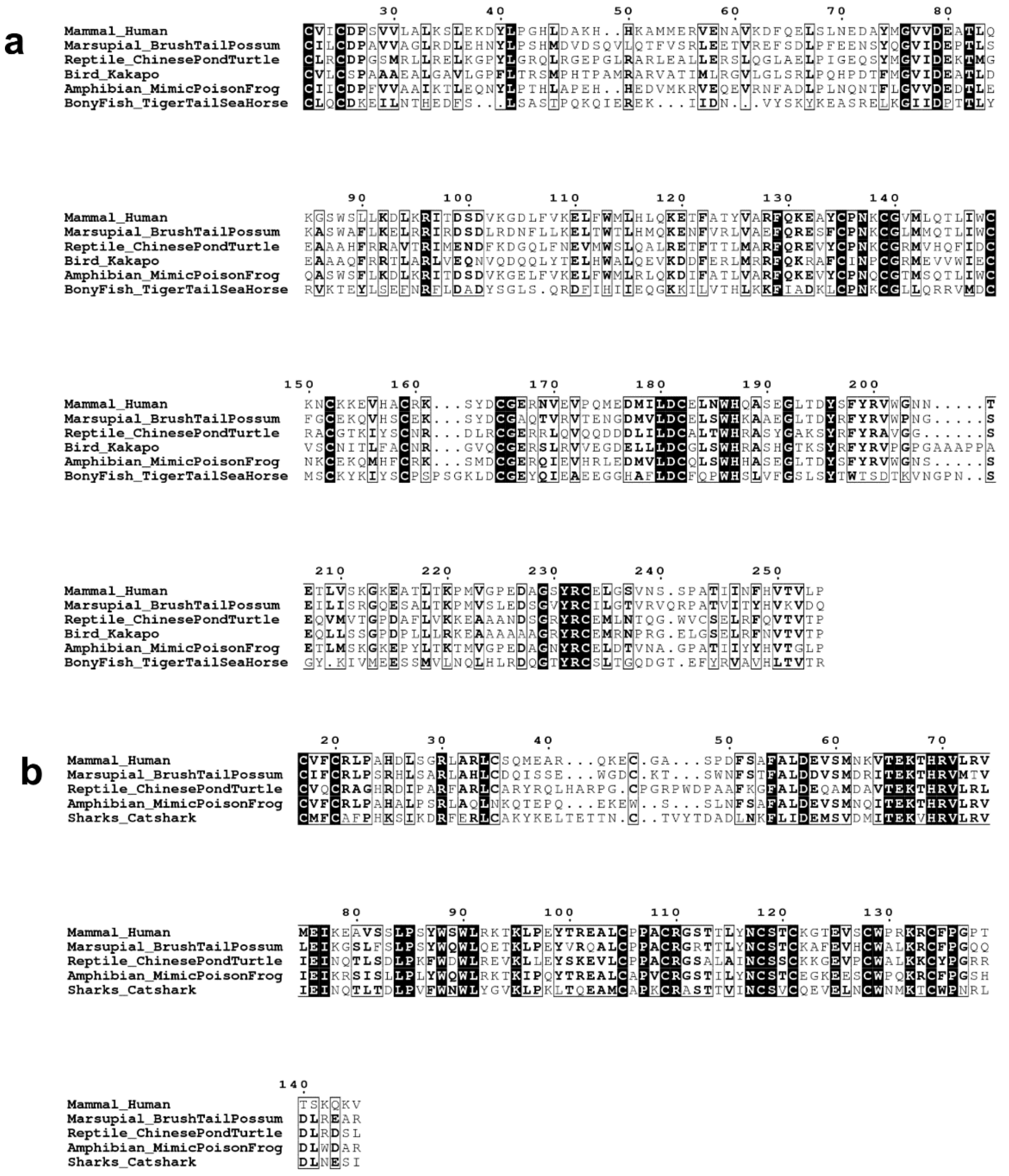

Figure S13. Sequence alignment of IZUMO1 and TMEM95 from multiple species. a Ectodomain sequences for IZUMO1 from human (*Homo sapiens*, NCBI: NP_872381), the marsupial brush tail possum (*Trichosurus vulpecula,* NCBI: XP_036602972), the Chinese pond turtle (*Mauremys reevesii,* NCBI: XP_039366991), the flightless parrot Kākāpō (*Strigops habroptila*, NCBI: XP_030366609), the mimic poison frog (*Ranitomeya imitator*, NCBI: CAF4957531), and the tiger tail seahorse (*Hippocampus comes*, NCBI XP_019744032) were aligned using CLUSTAL OMEGA. **b** Ectodomain sequences for TMEM95 from human (*Homo sapiens*, NCBI: XP_016880054), the marsupial brush tail possum (*Trichosurus vulpecula,* NCBI: XP_036622079), the Chinese Pond Turtle (*Mauremys reevesii,* NCBI: XP_039355851), the mimic poison frog (*Ranitomeya imitator*, NCBI: CAF5092145), and the catshark (*Scyliorhinus canicular*, NCBI: XP_038642792) were aligned using CLUSTAL OMEGA. Strictly conserved residues are displayed as white text on a black background. Residues with conservative substitutions are in bold. Both alignments are numbered according to the human sequence.

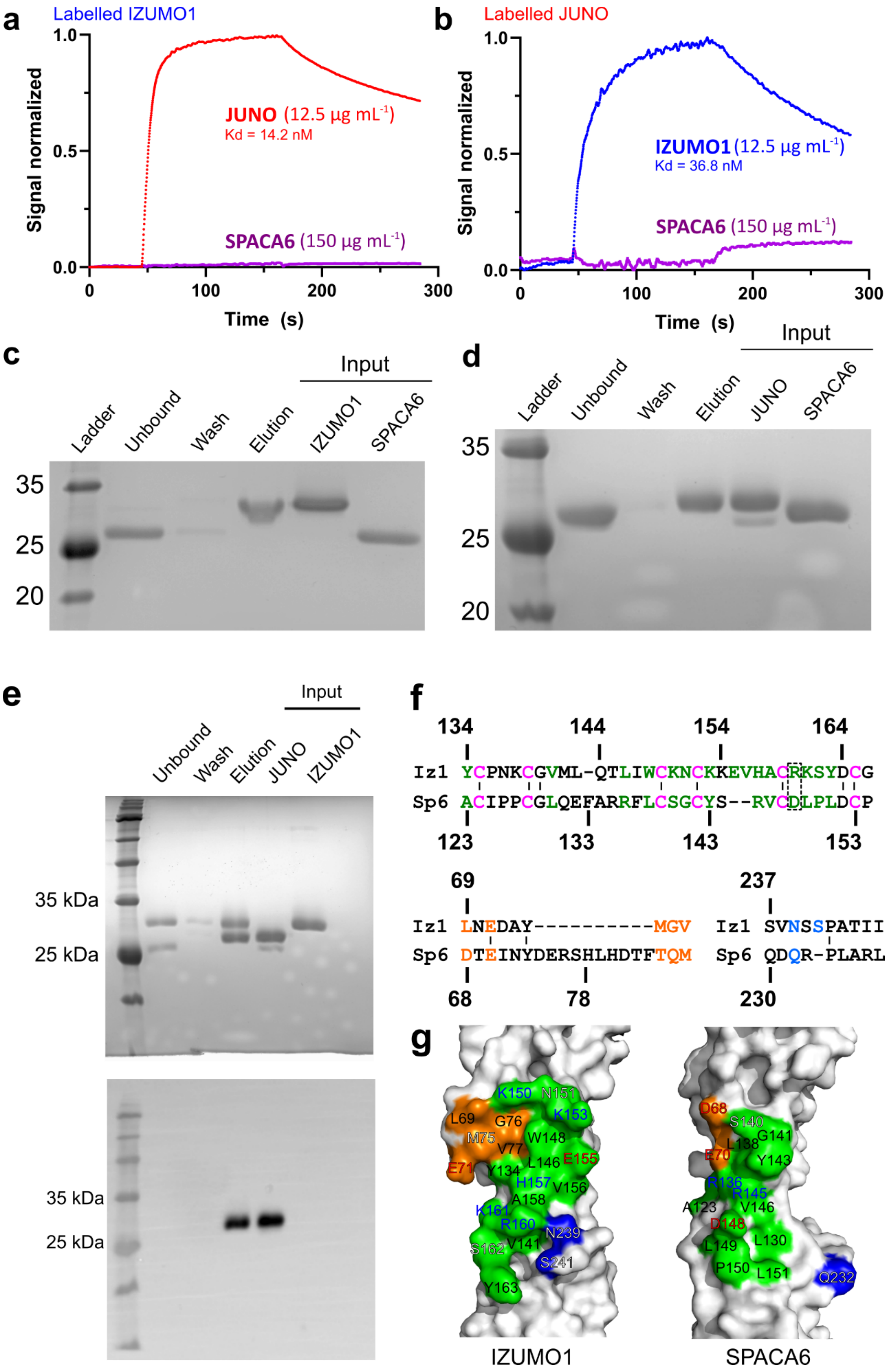
a
Labelled IZUMO1
Signal normalized
JUNO (12.5 μg mL-1)
Kd = 14.2 nM
SPACA6 (150 μg mL-1)
Time (s)
b
Labelled JUNO
Signal normalized
IZUMO1 (12.5 μg mL-1)
Kd = 36.8 nM
SPACA6 (150 μg mL-1)
Time (s)
c
Input
Ladder
Unbound
Wash
Elution
IZUMO1
SPACA6
35
25
20
d
Input
Ladder
Unbound
Wash
Elution
JUNO
SPACA6
35
25
20
e
Input
Unbound
Wash
Elution
JUNO
IZUMO1
35 kDa
25 kDa
35 kDa
25 kDa
f
134 144 154 164
Iz1 YCPNKCGVML-QTLIWCKNCKKEVHACRKSYDCG
Sp6 ACIPPCGLQEFARRFLCSGCYS--RVCDLPLDCP
123 133 143 153
69
Iz1 LNEDAY----------MGV
Sp6 DTEINYDERSHLHDTFTQM
68 78
237
Iz1 SVNSSPATII
Sp6 QDQR-PLARL
230
g
K150
N151
K153
L69
G76
M75
W148
V77
E71
Y134
L146
E155
H157
V156
A158
K161
R160
N239
S162
V141
S241
Y163
IZUMO1
D68
S140
G141
L138
E70
Y143
R136
R145
A123
V146
D148
L149
L130
P150
L151
Q232
SPACA6

Figure S14. SPACA6 does not bind to IZUMO1 and JUNO. BLI sensorgrams of biotin-labelled **a** IZUMO1 or **b** JUNO as bait with high concentrations of JUNO, IZUMO1 or SPACA6 proteins as the analyte. Pull-down analyses of His-tagged **c** IZUMO1 or **d** JUNO as bait with equal concentrations of untagged SPACA6 as analyte protein. SDS-PAGE analyses stained with Coomassie brilliant blue are used to detect the protein. Both BLI and pull-downs results are representative of independent duplicate (*n*=2) studies. **e** Positive control for pull-down assays. Coomassie stained SDS-PAGE (top) and Western blot of His-tagged JUNO incubated with untagged IZUMO1 (bottom). Mouse anti-His primary antibody was used with an HRP-conjugated anti-mouse secondary for detection of proteins. **f** Sequence alignments between the portions of IZUMO1 that interact with JUNO and their equivalent positions in SPACA6. IZUMO1 residues known to interact with JUNO are colored depending on the region in which they are found (green = hinge, orange = helix, blue = Ig-like). Cysteine residues are colored magenta. **g** Surface representation of the portion of IZUMO1 that interacts with JUNO and the equivalent position in SPACA6. Interacting surfaces are colored depending on the region in which they are found, as above. Residue names are colored depending on the biochemical characteristics of the residue (red = negatively charged, blue = positively charged, white = polar, black = non-polar).

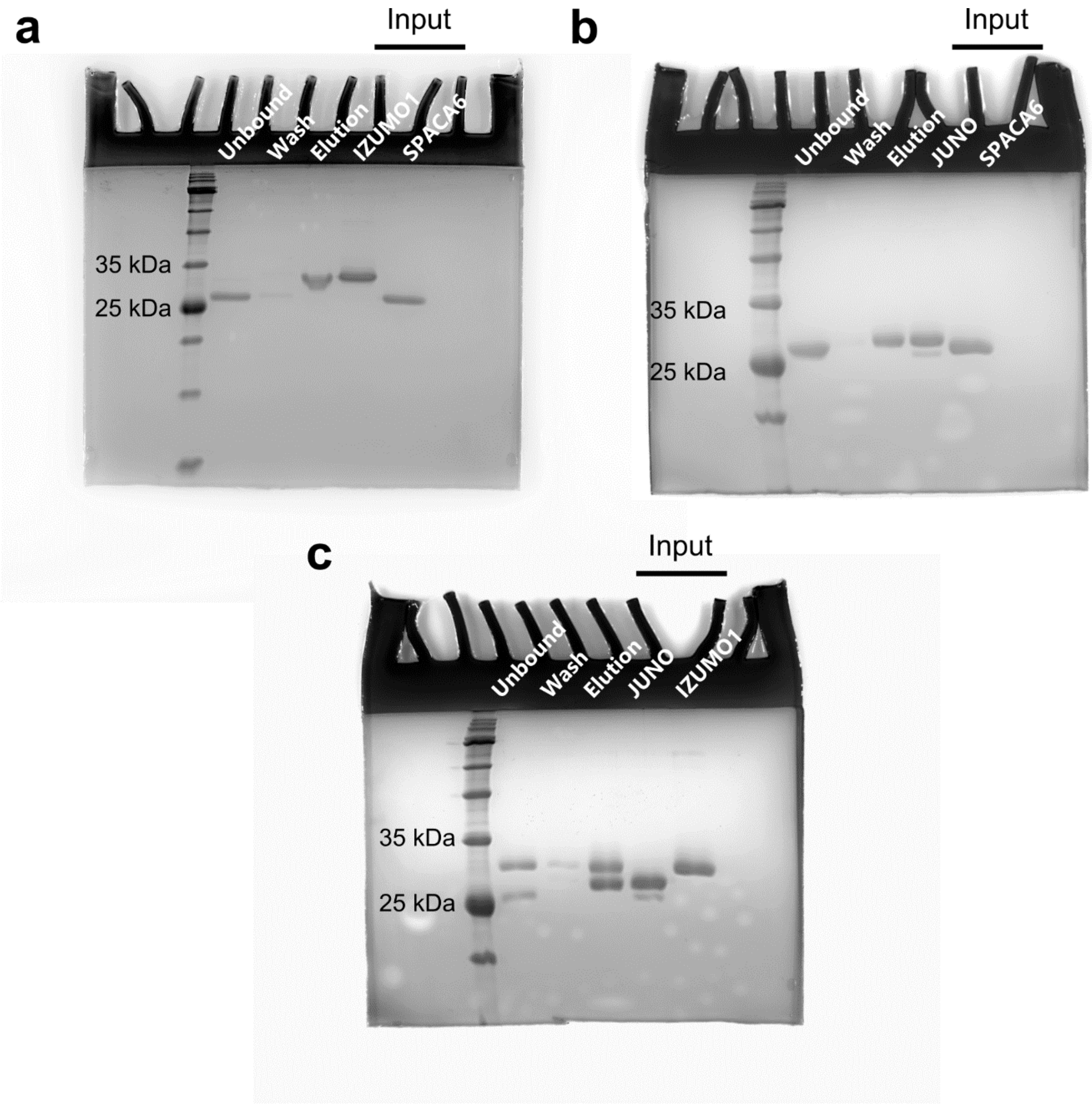

Figure S15. Source data for pull-down assays. Uncropped Coomassie stained SDS-PAGE source data for: **a** Supplementary Figure 14c, **b** Supplementary Figure 14d, and **c** Supplementary Figure 14e.

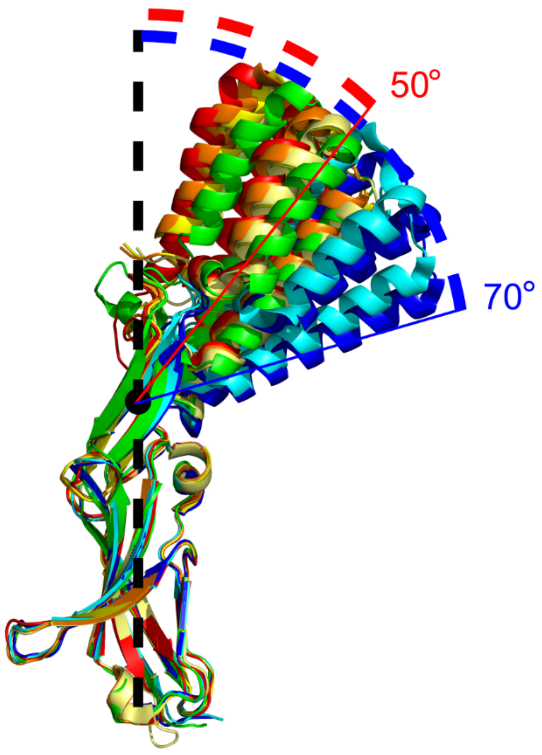

Figure S16. Crystallographic evidence of IZUMO1 flexibility. Alignments of the multiple IZUMO1 solved structures by the Ig-like domain to compare orientation of the 4HB; the smallest and largest angles between the bottom of the Ig-like domain, the center of the hinge region, and the tip of helix 4 in the 4HB are displayed. IZUMO1 structures include PDB: 5F4E (red), 5JKC (orange), 5JKD (yellow), 5JK9 (light yellow), 5B5K (green), 5F4T (cyan), and 5F4V (blue).

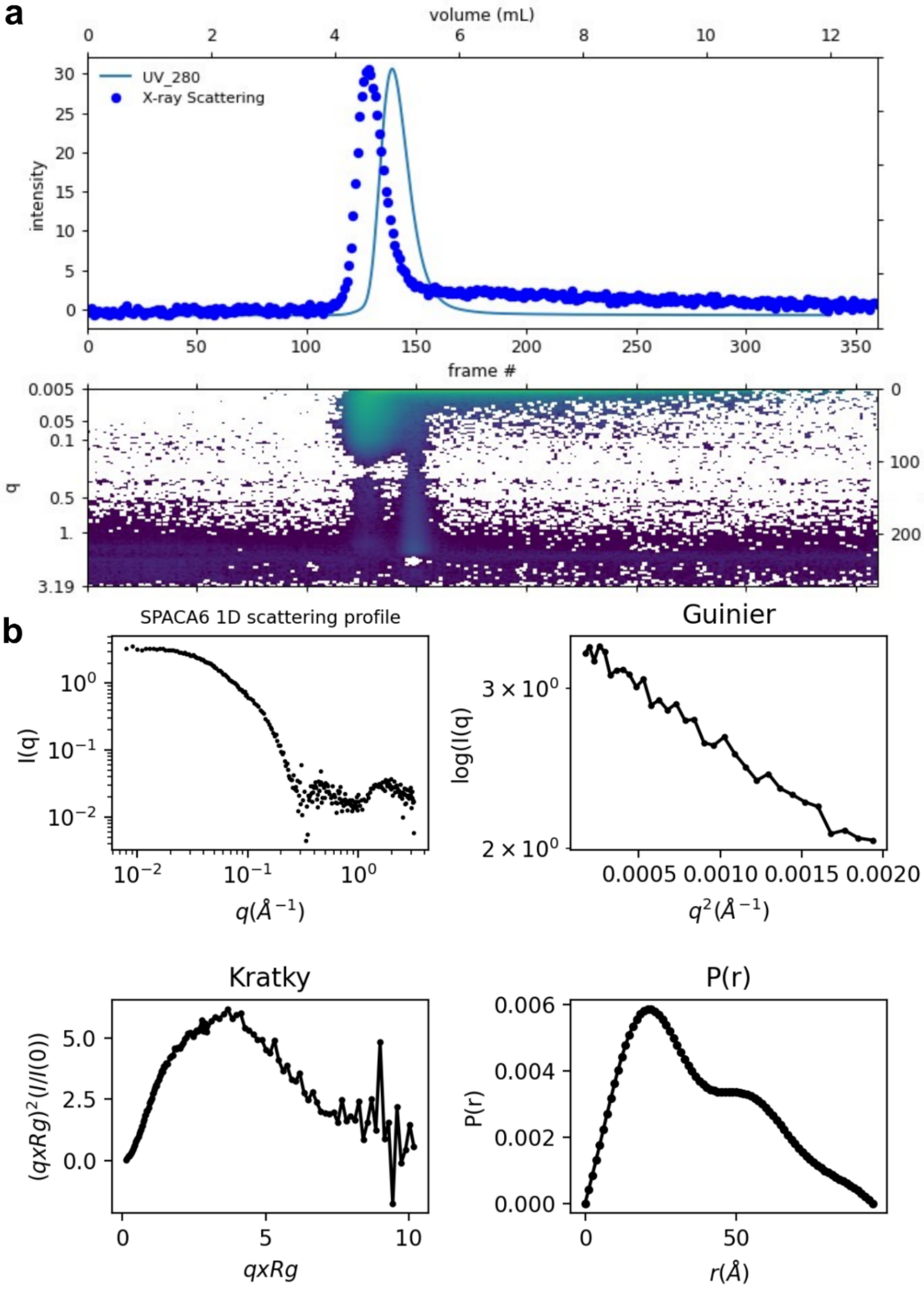

Figure S17. SEC-SAXS data of SPACA6. a Top panel shows X-ray scattering intensity (blue dots) and UV absorbance 280 nm trace (solid blue line) of SEC-SAXS elution profile (360 frames). Bottom panel represents a 2D heat map of the buffer subtracted X-ray intensity. Each vertical slice represents the X-ray intensity of the entire SAXS/WAXS q range from 0.005-3.1$Å^{-1}$ for each frame. **b** Average intensity of frames 90-100 was used to perform buffer subtraction on frames 122-125 of the SPACA6 elution peak. A R_g of 29.5 Å was calculated from the Guinier plot and D_{max} of 95 Å was determined from the Distance Distribution function (P(r)). Dimensionless Kratky plot indicates the protein is well folded but displays flexibility.

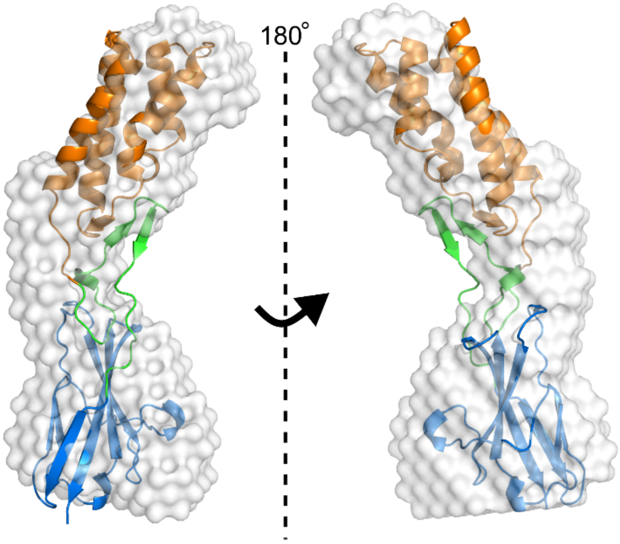

Figure S18. SAXS reconstruction of SPACA6. *Ab initio* reconstruction of SPACA6 ectodomain using SAXS date (white envelope) overlaid with the SPACA6 crystal structure, colored according to its three regions (4HB, orange; Ig-like domain, blue; hinge, green). DAMMIF was used to produce 20 envelope models, which were subsequently averaged with DAMAVER and filtered with DAMFILT.

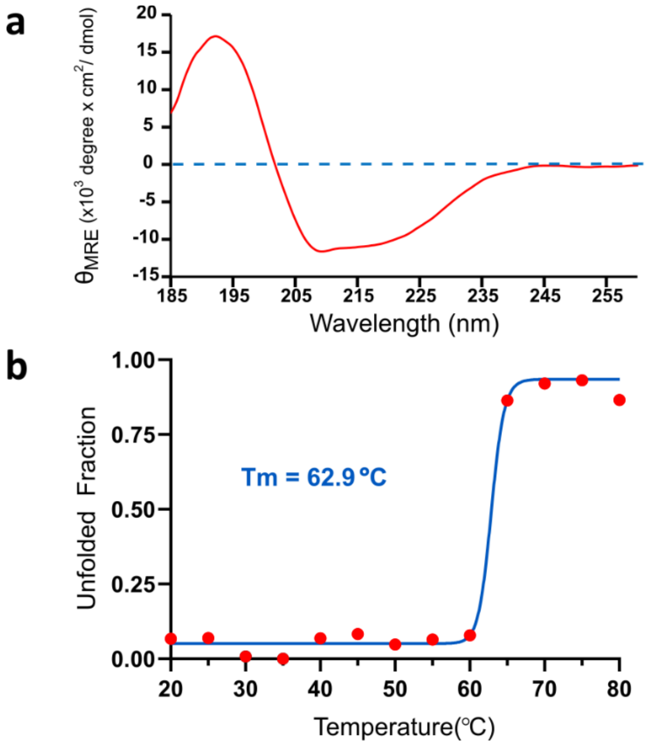

Figure S19. CD spectra and thermal melt of IZUMO1 ectodomain. a Far-UV CD spectra of the IZUMO1 ectodomain at 0.2 mg mL^{-1}. **b** Thermal melt of IZUMO1 ectodomain in 10 mM sodium phosphate, pH 7.4, and 150 mM NaF.

Table S1. Dali server search of PDB.

Protein Name	PDB	Z-score	RMSD	LALI	%ID
**Full Ectodomain***					
IZUMO SPERM-EGG FUSION PROTEIN 1	5jk9-A	12.1	3.9	130	15
DOWN SYNDROME CELL ADHESION MOLECULE	3dmk-A	9.2	6.7	92	13
MUCOSA-ASSOCIATED LYMPHOID TISSUE TRANSLOCATION PROTEIN	3bfo-A	8.8	1.7	75	15
COXSACKIE AND ADENOVIRUS RECEPTOR	3j6n-K	8.6	2.3	81	21
CELL SURFACE GLYCOPROTEIN CD200 RECEPTOR 1	4bfi-A	8.6	2.8	82	17
LEUCINE-RICH REPEAT AND IMMUNOGLOBULIN-LIKE DOMAIN	4oqt-A	8.6	13.4	118	14
INTERLEUKIN-33	5vi4-F	8.5	3.7	97	9
BASIC FIBROBLAST GROWTH FACTOR RECEPTOR 1	2cr3-A	8.5	3	80	18
CD2	1hng-A	8.3	2.2	78	13
ADVANCED GLYCOSYLATION END PRODUCT-SPECIFIC RECEPTOR	4lp5-A	8.2	2.7	80	15
**Four-Helix Bundle†**					
IZUMO SPERM-EGG FUSION PROTEIN 1	5jk9-A	7.2	3.5	116	8
DESIGNED HELICAL REPEAT PROTEIN	5cwc-A	6.5	2.9	86	14
PHOSPHOPROTEIN	3l32-B	6.2	1.2	43	5
55-KDA IMMEDIATE-EARLY PROTEIN 1	6tgz-F	6	5	95	7
DOLICHYL-DIPHOSPHOOLIGOSACCHARIDE PROTEIN	6s7o-E	5.7	3.7	66	9
SOLUBLE CYTOCHROME B562	4or2-B	5.5	4.5	68	7
GLUTAMYL-TRNA REDUCTASE 1	5che-A	5.5	6.7	62	3
DNA-DEPENDENT RNA POLYMERASE SUBUNIT RPO132	6rfl-C	5.5	7.5	56	4
SPOROZOITE MICRONEME PROTEIN	4u5a-B	5.5	3.2	90	9
RHUL123	4wid-A	5.4	7	94	7
**Ig-like domain‡**					
IZUMO SPERM-EGG FUSION PROTEIN 1	5jk9-A	11.9	3.5	113	18
DOWN SYNDROME CELL ADHESION MOLECULE	3dmk-A	9.2	4.4	89	12
INTERFERON ALPHA-5	3oq3-B	8.7	2.5	88	15
COXSACKIE AND ADENOVIRUS RECEPTOR	3j6n-K	8.6	2.4	81	20
MUCOSA-ASSOCIATED LYMPHOID TISSUE TRANSLOCATION PROTEIN	3bfo-A	8.6	1.7	74	15
BASIC FIBROBLAST GROWTH FACTOR RECEPTOR 1	2cr3-A	8.5	4.1	83	17
LEUCINE-RICH REPEAT AND IMMUNOGLOBULIN-LIKE DOMAIN	4oqt-A	8.5	8.5	88	17
CELL SURFACE GLYCOPROTEIN CD200 RECEPTOR 1	4bfi-A	8.5	2.9	81	20
TYROSINE-PROTEIN PHOSPHATASE	4yh7-B	8.5	2.5	89	18
IP13724P	6qp8-A	8.3	2.7	101	11

* SPACA6 ectodomain structure, residues 27-246, was used to search the PDB25 subset of the Protein Data Bank.
† SPACA6 4HB + Hinge structure, residues 27-150, was used to search the PDB25 subset of the Protein Data Bank.
‡ SPACA6 Hinge + Ig-like domain structure, residues 121-246, was used to search the PDB25 subset of the Protein Data Bank.
