# Supplementary material for: SPACA6 ectodomain structure reveals a conserved superfamily of gamete fusion-associated proteins
Source: Commun Biol. 2022 Sep 17;5:984. doi: 10.1038/s42003-022-03883-y (PMC9482655; doi:10.1038/s42003-022-03883-y)
Supplement: Supplementary file 3 — Reporting summary [file 42003_2022_3883_MOESM3_ESM.pdf]

nature portfolio

Corresponding author(s): Jeffrey E. Lee

Last updated by author(s): Aug 8, 2022

Reporting Summary

Nature Portfolio wishes to improve the reproducibility of the work that we publish. This form provides structure for consistency and transparency in reporting. For further information on Nature Portfolio policies, see our Editorial Policies and the Editorial Policy Checklist.

Statistics

For all statistical analyses, confirm that the following items are present in the figure legend, table legend, main text, or Methods section.

n/a	Confirmed	
☐	☒	The exact sample size (*n*) for each experimental group/condition, given as a discrete number and unit of measurement
☐	☒	A statement on whether measurements were taken from distinct samples or whether the same sample was measured repeatedly
☒	☐	The statistical test(s) used AND whether they are one- or two-sided<br>*Only common tests should be described solely by name; describe more complex techniques in the Methods section.*
☒	☐	A description of all covariates tested
☒	☐	A description of any assumptions or corrections, such as tests of normality and adjustment for multiple comparisons
☐	☒	A full description of the statistical parameters including central tendency (e.g. means) or other basic estimates (e.g. regression coefficient) AND variation (e.g. standard deviation) or associated estimates of uncertainty (e.g. confidence intervals)
☒	☐	For null hypothesis testing, the test statistic (e.g. *F*, *t*, *r*) with confidence intervals, effect sizes, degrees of freedom and *P* value noted<br>*Give P values as exact values whenever suitable.*
☒	☐	For Bayesian analysis, information on the choice of priors and Markov chain Monte Carlo settings
☒	☐	For hierarchical and complex designs, identification of the appropriate level for tests and full reporting of outcomes
☒	☐	Estimates of effect sizes (e.g. Cohen's *d*, Pearson's *r*), indicating how they were calculated

Our web collection on statistics for biologists contains articles on many of the points above.

Software and code

Policy information about availability of computer code

Data collection	N/A
Data analysis	N/A

For manuscripts utilizing custom algorithms or software that are central to the research but not yet described in published literature, software must be made available to editors and reviewers. We strongly encourage code deposition in a community repository (e.g. GitHub). See the Nature Portfolio guidelines for submitting code & software for further information.

Data

Policy information about availability of data

All manuscripts must include a data availability statement. This statement should provide the following information, where applicable:

- Accession codes, unique identifiers, or web links for publicly available datasets
- A description of any restrictions on data availability
- For clinical datasets or third party data, please ensure that the statement adheres to our policy

Further information and requests for resources and reagents are available from the corresponding author on reasonable requests. Atomic coordinates and structure factors for SPACA6 have been deposited in the Protein Data Bank (PDB) with the accession code: 7TA2. SAXS data have been deposited in the Small Angle Scattering Biological Data Bank (SASBDB) with the accession code: SASDNM3. Source data are provided with this paper.

Field-specific reporting

Please select the one below that is the best fit for your research. If you are not sure, read the appropriate sections before making your selection.

☒ Life sciences ☐ Behavioural & social sciences ☐ Ecological, evolutionary & environmental sciences

For a reference copy of the document with all sections, see nature.com/documents/nr-reporting-summary-flat.pdf

Life sciences study design

All studies must disclose on these points even when the disclosure is negative.

Sample size	Due to the biophysical nature of our work under controlled settings with minimal variables, sample sizes of 3 were considered sufficient.
Data exclusions	No data was excluded
Replication	Replicates from distinct protein preps were undertaken to confirm reproducibility. In all cases, data was reproducible.
Randomization	N/A
Blinding	N/A

Reporting for specific materials, systems and methods

We require information from authors about some types of materials, experimental systems and methods used in many studies. Here, indicate whether each material, system or method listed is relevant to your study. If you are not sure if a list item applies to your research, read the appropriate section before selecting a response.

Materials & experimental systems

n/a	Involved in the study	
☐	☒	Antibodies
☐	☒	Eukaryotic cell lines
☒	☐	Palaeontology and archaeology
☒	☐	Animals and other organisms
☒	☐	Human research participants
☒	☐	Clinical data
☒	☐	Dual use research of concern

Methods

n/a	Involved in the study	
☒	☐	ChIP-seq
☒	☐	Flow cytometry
☒	☐	MRI-based neuroimaging

Antibodies

Antibodies used	Anti-His Tag Primary from Mouse (Roche, cat. #11922416001, lot #37569600) + Anti-mouse IgG (H+L) antibody from Goat (Invitrogen, cat. #62-6520, lot #SA248187)
Validation	Anti-His primary was validated by manufacturer's (Roche), with the lot# used confirmed as "meeting our specification" in the Certificate of Analysis.

Eukaryotic cell lines

Policy information about cell lines

Cell line source(s)	Drosophila S2
Authentication	Cells are commercially available from Gibco. Here, they are used solely for the production of recombinant protein.
Mycoplasma contamination	These cells were not tested for mycoplasma contamination.
Commonly misidentified lines (See ICLAC register)	N/A
